# Lymphocyte-to-Monocyte Ratio Is Independently Associated with Progressive Infarction in Patients with Acute Ischemic Stroke

**DOI:** 10.1155/2022/2290524

**Published:** 2022-12-27

**Authors:** Xiaocheng Mao, Qiulong Yu, Yunfang Liao, Qin Huang, Si Luo, Shumeng Li, Yuexin Qiu, Yuhang Wu, Jinchong Zhang, Qianxi Chen, Min Zhu, Huihua Li, Jing Lin, Daojun Hong

**Affiliations:** ^1^Department of Neurology, The First Affiliated Hospital of Nanchang University, Nanchang, 330000 Jiangxi, China; ^2^Department of Pharmacy, The First Affiliated Hospital of Nanchang University, Nanchang, 330000 Jiangxi, China; ^3^Jiangxi Province Key Laboratory of Preventive Medicine, School of Public Health, Nanchang University, Nanchang, 330000 Jiangxi, China

## Abstract

**Methods:**

From April 2017 to December 2020, we retrospectively recruited 477 patients with acute ischemic stroke (within 48 hours after onset). Progressive infarction was defined as an increase of ≥1 point in motor power or ≥2 points on the total National Institutes of Health Stroke Scale (NIHSS) within 7 days after admission and extension of the original infarction were further confirmed by diffusion-weighted imaging. Demographic characteristics, clinical information, and neuroimaging characteristics were evaluated after admission. All blood draws and initial imaging were completed within 24 hours of admission.

**Results:**

PI occurred in 147 (30.8%) patients. Univariate analysis comparing the two groups revealed that hypertension, initial NIHSS score, discharge NIHSS score, modified Rankin scale score at 90 days, monocyte level, creatinine level, fasting glucose level, LMR, monocyte-to-high-density lipoprotein ratio (MHR), and lesion location were significantly different (*P* < 0.05). Multivariate logistic regression analysis showed that the odds ratio of PI increased as the quartile of LMR increased, with the lowest quartile as the reference value. Subgroup analyses showed that a high LMR was an independent predictor of PI only in large artery atherosclerosis (LAA) patients. The receiver operating characteristic (ROC) curve was drawn to estimate the predictive value of LMR for PI. For all cases, the area under the curve was 0.583 (95% CI 0.526-0.641), and the best predictive cutoff value was 3.506, with a sensitivity of 53.1% and a specificity of 63.9%. In patients with LAA, the area under the curve was 0.585 (95% CI 0.505-0.665), and the best predictive cutoff value was 3.944, with a sensitivity of 48.7% and a specificity of 72.8%.

**Conclusions:**

LMR was an independent predictor for progressive infarction in patients with acute ischemic stroke, especially in LAA cerebral infarction patients.

## 1. Introduction

Ischemic stroke is a global disease and the leading cause of long-term disability in adults [1], and China faces the greatest challenge from stroke in the world. The Global Burden of Disease Study (GBD) 2019 estimated that deaths caused by stroke in China reached approximately 1.57 million in 2018 [2]. Approximately one-third of patients with acute ischemic stroke (AIS) experience early neurological deterioration (END) with poor outcomes [3]. END, a symptomatic diagnosis, has been defined as an increase of ≥1 point in motor power or ≥2 points in the total National Institute of Health Stroke Scale (NIHSS) score within 7 days after admission. However, various mechanisms are involved in END, including progressive infarction (PI) representing extension of the original infarction, increased intracranial pressure, recurrent cerebral ischemia on behalf of new stroke, and secondary parenchymal hemorrhage [3–5]. In recent years, many predictors have been associated with END, such as the initial National Institute of Health Stroke Scale (NIHSS) score [6], the triglyceride-glucose index [7], high-sensitivity C-reactive protein [8], albuminuria [9], and fibrinogen [10].

Recently, the lymphocyte-to-monocyte ratio (LMR), as a surrogate marker of systemic inflammation, and its high or low value, represents the degree of inflammatory response [11, 12]. LMR has been found to be associated with poor prognosis in various malignancies [13, 14]. Quan et al. found that the LMR value might be associated with higher short-term and long-term mortality in acute coronary syndrome patients [15]. In studies of AIS, LMR at admission was independently associated with poor 3-month outcome [12] and higher risk for hemorrhagic transformation in patients with AIS [16]. A study by Gong et al. [17] showed that LMR was associated with postthrombolysis END. However, to the best of our knowledge, there are few studies on the relationship between LMR and PI.

PI is the most common cause of END in acute ischemic stroke, accounting for approximately 30% of cases [4]. Since LMR has attracted increasing attention in recent years, the aim of this work is to investigate the relationship between LMR and PI.

## 2. Methods

### 2.1. Patients

We retrospectively collected inpatients diagnosed with AIS in the Stroke Unit of First Affiliated Hospital of Nanchang University between April 2017 and December 2020. All procedures were approved by the Ethics Committee of First Affiliated Hospital of Nanchang University. Patients were recruited if they met the following criteria: (1) admission to our hospital within 48 hours after symptom onset and (2) evidence of cerebral infarction on diffusion-weighted imaging (DWI) consistent with the clinical deficit. Patients were excluded from this study if they (1) were lacking complete imaging, laboratory, and follow-up data, (2) had fever or infection on admission or had a history of immune system diseases, or (3) had received intravenous thrombolysis or endovascular therapy.

### 2.2. Clinical Characteristics and Laboratory Parameters

Demographic characteristics and clinical information, including age, sex, history of hypertension and diabetes, initial NIHSS score, discharge NIHSS score, and modified Rankin scale (mRS) score at 90 days, were recorded. The mRS was evaluated in person or over the telephone. We mainly included laboratory variables based on the following two points. On the one hand, we included most basic laboratory indicators. On the other hand, we collected variables related to END reported in the previous studies. Finally, the levels of white blood cells, red blood cells, hemoglobin, blood platelets, lymphocytes, monocytes, neutrophilic granulocytes, fibrinogen, D-dimer, blood urea nitrogen, creatinine, uric acid, total cholesterol, triglycerides, high-density lipoprotein cholesterol, low-density lipoprotein cholesterol, LMR, monocyte-to-high-density lipoprotein ratio (MHR), neutrophil-to-lymphocyte ratio (NLR), and platelet-to-lymphocyte ratio (PLR) were collected within 24 hours of admission.

### 2.3. Evaluation of Neuroimaging Information

All patients underwent magnetic resonance imaging (MRI) in a 3.0-Tesla scanner within 48 hours of onset and immediately after neurological deterioration was detected. The protocol included T1-weighted, T2-weighted, fluid-attenuated inversion recovery (FLAIR), DWI (TR/TE 3100/91 ms; field of view 230 × 230 mm^2^; 19 slices with slice thickness 5 mm; voxel size = 1.2 × 1.2 × 5 mm^3^; 2b values of 0 and 1000 s/mm^2^; scan time 1.16 min), and three-dimensional time-of-flight magnetic resonance angiography (TR/TE 22/3.86 ms; field of view 235 × 235 mm^2^; voxel size 0.9 × 0.6 × 0.6 mm^3^; 2b values of 0 and 1000 s/mm^2^; scan time 3.12 min). All images were reviewed and evaluated by two trained neurologists who were blinded to the patients' information. Leukoaraiosis was analyzed by a 4-point score as proposed by Fazekas et al. [18]. To determine the subtype of ischemic stroke, [19] criteria were used [20].

### 2.4. Definition of PI

Progressive infarction (PI) was defined as an increase of ≥1 point in motor power or ≥2 points on the total NIHSS within 7 days after admission and extension of the original infarction were further confirmed by diffusion-weighted imaging [21]. All patients were grouped according to the definition of PI after careful case review by a stroke physician.

### 2.5. Statistical Analysis

All statistical analyses were performed using SPSS version 26.0 (SPSS Inc.). Baseline characteristics and risk factors were compared using Student's *t*-test or the Mann–Whitney *U* test for continuous variables and the chi-square test or Fisher's exact test for categorical variables. Continuous variables with a normal distribution are presented as mean ± SD, variables with a nonnormal distribution are expressed as median (interquartile range (IQR)), and categorical variables are presented as frequency (percentage). For all cases, we used multivariate logistic regression analysis to detect the risk factors for PI. Based on the LMR values, we grouped patients according to the interquartile range principle, and the lowest quartile was used as the reference (all patients were divided into quartiles based on LMR. Q1, <2.2327; Q2, 2.2327-3.2041; Q3, 3.2041-4.3103; and Q4, >4.3103). Subsequently, the association between LMR and PI was analyzed among several subgroups according to TOAST with the use of logistic regression models. We only performed subgroup analysis on large artery atherosclerosis (LAA) patients and small vessel occlusion (SVO) patients, on the one hand, because these two groups of patients were the most common and, on the other hand, because the sample size of other subgroups was small. In the LAA subgroup, the patients were divided into quartiles based on LMR (Q1, <2.4250; Q2, 2.4250-3.3514; Q3, 3.3514-4.50; and Q4, >4.50). In the SVO subgroup, the patients were divided into quartiles based on LMR (Q1, <2.6906; Q2, 2.6906-3.5762; Q3, 3.5762-4.7133; and Q4, >4.7133). SPSS software was used to generate receiver operating characteristic (ROC) curves to evaluate the ability of the LMR to predict PI and to determine its specificity, sensitivity, and optimal cutoff point. Significance levels were set at *P* < 0.05 for 2-tailed tests.

## 3. Results

**Figure 1:**
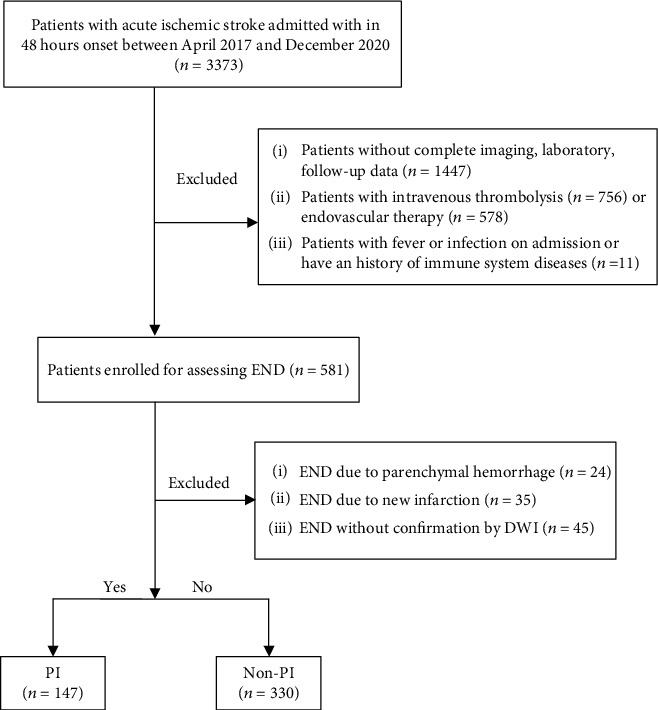
Selection of study participants.

From April 2017 to December 2020, 3373 AIS patients admitted within 48 hours onset were screened according to inclusion criteria and exclusion criteria (Figure 1), and a total of 477 eligible patients were enrolled, including 147 (30.8%) in the PI group and 330 (69.2%) in the non-PI (nonprogressive infarction) group. The baseline data of the two groups are listed in Table 1. We found that hypertension, initial NIHSS score, discharge NIHSS score, mRS score at 90 days, monocyte level, creatinine level, fasting glucose level, LMR, MHR, and lesion location were significantly different between groups (*P* < 0.05). There were no differences in age, sex, history of diabetes, white blood cell count, red blood cell count, platelets, total cholesterol, triglycerides, high-density lipoprotein cholesterol, low-density lipoprotein cholesterol, fibrinogen, D-dimer, TOAST classification, or leukoaraiosis between the two groups (*P* > 0.05).

As shown in Table 2, the highest quartile of LMR (the first quartile as the reference value) was independently associated with PI, as demonstrated by multivariate logistic regression analysis done after grouping LMR by quartile. The multivariable logistic regression model adjusted for age and sex revealed that the highest LMR quartiles were independently associated with PI (OR 2.144, 95% CI 1.211-3.796, *P* = 0.009). The second adjustment for hypertension, initial NIHSS score, creatinine level, fasting glucose level, and lesion location also suggested that a high LMR was independently associated with PI (OR 2.362, 95% CI 1.326-4.208, *P* = 0.004). After adjusting for confounding factors such as hypertension, initial NIHSS score, creatinine level, blood glucose level, lesion location, blood urea nitrogen level, uric acid level, TOAST classification, and leukoaraiosis, LMR was still significantly associated with PI (OR 2.598, 95% CI 1.405-4.802, *P* = 0.002). The results of the subgroup analyses are shown in Table 3. LMR was an independent risk factor for PI in patients with LAA (OR 2.335, 95% CI 1.051-5.191, *P* = 0.037). It had no correlation in patients with SVO (*P* > 0.05).

**Figure 2:**
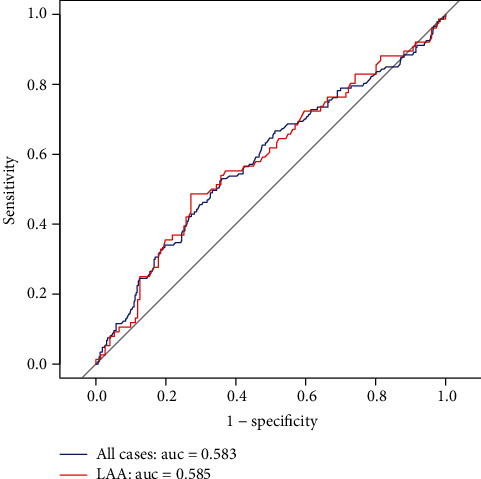
Receiver operating characteristic curve for LMR to predict progressive infarction in all cases and LAA patients. LMR: lymphocyte to monocyte ratio; LAA: large artery atherosclerosis; AUC: area under the curve.

The receiver operating characteristic (ROC) curve was drawn to estimate the predictive value of LMR on PI (Figure 2). For all cases, we observed that the area under the curve was 0.583 (95% CI 0.526-0.641), and the best predictive cutoff value was 3.506, with a sensitivity of 53.1% and a specificity of 63.9%. In patients with LAA, the area under the curve was 0.585 (95% CI 0.505-0.665), and the best predictive cutoff value was 3.944, with a sensitivity of 48.7% and a specificity of 72.8%. When we divided patients into two groups around the cutoff value (Table 4), the frequency of PI was 24.6% in patients with LMR < 3.506 and 39.6% in patients with LMR ≥ 3.506 (*P* < 0.001). Of note, after dividing LAA patients into two groups around their optimal cutoff value of 3.944, the analysis demonstrated that the frequency of PI was 26.2% in patients with LMR < 3.944 and 47.4% in patients with LMR ≥ 3.944 (*P* = 0.001).

## 4. Discussion

END occurs in approximately one-third of AIS patients. The definition of END is based on symptomatic changes (an increase of ≥1 point in motor power or ≥2 points in the total NIHSS score within 7 days after admission) [22]. However, causes of END include PI representing extension of the original infarction, increased intracranial pressure, recurrent cerebral ischemia on behalf of new stroke, and secondary cerebral parenchymal hemorrhage. Among them, PI accounts for approximately 33.6% of END cases [4]. In our study, we purely focused on the relationship between LMR and PI and defined progressive infarction as an increase of ≥1 point in motor power or ≥2 points on the total NIHSS within 7 days after admission but the enlargement of the original infarction was confirmed by diffusion-weighted imaging. In our study, we purely focused on the association between serum inflammatory factors and PI and found that LMR was a predictor of PI in AIS patients, especially in patients with LAA. In the whole cohort, patients with LMR ≥ 3.506 were more likely to develop PI, and LMR ≥ 3.944 increased the risk of PI in patients with LAA.

Among the lymphocytes, T lymphocytes have a damaging role in the process of stroke [23]. In this subgroup of cells, type 1 helper T cells (Th1) are proinflammatory, and type 2 helper T cells (Th2) are anti-inflammatory [24]. Some studies have also reported the damaging effect of double-negative T lymphocytes in stroke and the protective effect of regulatory T cells on nerves [25, 26]. Although the role of lymphocytes in stroke is controversial, decreased lymphocyte counts after stroke have been demonstrated [27, 28], which is consistent with the results of this study. Monocytes have a pivotal role in the systemic inflammatory response; when they leave the circulatory system to reach tissues, they can differentiate into macrophages to play an inflammatory role [29]. The monocytes present during cerebral ischemia mainly come from the spleen, and the spleen-derived monocytes are also the first to enter the ischemic focus [30]. This finding was also confirmed in rats with middle cerebral artery occlusion by Seifert et al. [31]. The peripheral blood mononuclear cell count may be reduced due to the rapid recruitment of monocytes to the location of cerebral ischemia. Traditional monocytes fall into three categories: classic monocytes (CD14^hi^CD16^−^ monocytes), non-classic monocytes (CD14^dim^CD16^+^ monocytes), and intermediate monocytes (CD14^hi^CD16^+^ monocytes) [32–34]. Studies have reported that the levels of different types of monocytes increase at different time points after stroke, intermediate monocytes are the main monocytes in PI, and their levels begin to increase 3-16 days after onset [35]. This finding provides evidence for a decrease in peripheral blood mononuclear count within 24 hours after the onset of progressive infarction.

Recent studies have reported that low LMR values have an impact on the outcome and prognosis of stroke patients [12, 36]. Cai et al. found that a lower LMR at admission was independently associated with long-term all-cause mortality and major adverse cardiac events after discharge in patients with ST-elevation myocardial infarction [37]. However, the odds of cardiovascular events in patients with early coronary heart disease manifestations were the highest in patients with an elevated LMR [38]. Lombardi et al. also found that a higher LMR is directly associated with any type of clinical diagnosis of dementia [39]. Additionally, our study confirmed that a higher LMR value was an independent risk factor for PI. Of note, some studies on the correlation between LMR and END have used END as an outcome variable, which may reduce the specificity of the predictor. In our study, we focused only on the association between LMR and PI.

There are different mechanisms of ischemic stroke, and they are associated with different incidences of END [40]. According to the TOAST classification, patients with ischemic stroke caused by noncardiac embolism are more likely to worsen than patients with cardioembolic infarction [41]. However, no studies have reported the effect of LMR on AIS classification. Atherosclerosis is an inflammatory disease, and intensified inflammatory activation may lead to local proteolysis, plaque rupture, and thrombus formation, which causes ischemia and infarction [42]. However, the pathogenesis of ischemic stroke in SVO is mostly caused by small vessel disease (lipohyalinosis or fibrinoid necrosis) and has no obvious relationship with the occurrence and development of inflammatory diseases [43]. Therefore, LMR, as a novel biomarker of systemic inflammation, may have a greater impact on LAA patients. Then, we analyzed the relationship between LMR and PI in patients with different mechanisms or different lesion topographies and found that LMR was an independent predictor of PI only in patients with LAA cerebral infarction.

This study has some limitations. First, this was a single-center retrospective study of patients of a single ethnic background. Second, few patients had cardioembolism, undetermined etiology, or other etiology under the TOAST classification, so we only analyzed the relationship between LMR and LAA-type and small-artery-occlusion-type PI patients. Subsequent studies need to further expand the sample size and collect multicenter and multiethnic patients to confirm the findings. Finally, we only recorded the LMR from the first blood draw and did not monitor the LMR for the rest of the hospital stay or at discharge. There is some controversy about the relationship between LMR levels and END, so further research needs to continuously monitor the LMR value to determine whether the LMR is increased or decreased in END patients.

In conclusion, LMR was an independent predictor of progressive infarction in patients with acute ischemic stroke, especially LAA cerebral infarction. What is more, patients with progressive infarction tend to have worse outcome. Therefore, it is important to identify the predictors for progressive infarction.

## Figures and Tables

**Table 1:** Comparison of baseline characteristics between PI and non-PI groups.

Variable	PI (*n* = 147)	Non-PI (*n* = 330)	*P*
Demographic characteristics			
Age	63.79 ± 11.75	63.63 ± 11.99	0.895
Male, *n* (%)	90 (61.2%)	220 (66.7%)	0.25
Hypertension, *n* (%)	93 (63.3%)	172 (52.1%)	**0.028** ^∗^
Diabetes, *n* (%)	44 (29.9%)	75 (22.7%)	0.109
Initial NIHSS, median (IQR)	3.0 (2, 6)	2.5 (1, 6)	**0.011** ^∗^
Laboratory data			
WBC (10^∗^9/L), median (IQR)	7.13 (5.92, 8.88)	7.02 (5.79, 8.68)	0.398
RBC (g/L), median (IQR)	4.43 (4.01, 4.78)	4.4 (4.04, 4.77)	0.86
HGB (g/L), median (IQR)	133.0 (119, 145)	133.0 (122, 145)	0.384
PLT (10^∗^9/L), median (IQR)	209.0 (162, 249)	205.0 (167, 252)	0.61
Lymphocyte (10^∗^9/L), median (IQR)	1.46 (1.02, 1.96)	1.49 (1.10, 1.85)	0.55
Monocyte (10^∗^9/L), median (IQR)	0.42 (0.32, 0.55)	0.48 (0.37, 0,63)	**0.001** ^∗^
Neutrophilic granulocyte (10^∗^9/L), median (IQR)	4.92 (3.87, 6.89)	4.72 (3.55, 6.19)	0.132
TP (g/L), median (IQR)	65.42 (61.0, 69.3)	65.42 (61.9, 68.8)	0.802
BUN (mmol/L), median (IQR)	5.1 (3.80, 6.40)	4.9 (3.90, 6.53)	0.622
Cr (umol/L), median (IQR)	65.1 (53.7, 79.9)	68.6 (58.1, 83.83)	**0.041** ^∗^
Uric acid (mmol/L), median (IQR)	308.0 (235, 391.9)	318.5 (264, 392)	0.124
Fasting glucose (mmol/L), median (IQR)	6.39 (5.27, 8.38)	5.67 (4.80, 7.12)	**0.001** ^∗^
Total cholesterol (mmol/L), median (IQR)	4.38 (3.65, 5.08)	4.38 (3.59, 4.97)	0.611
Triglyceride (mmol/L), median (IQR)	1.36 (0.99, 1.98)	1.37 (0.92, 1.79)	0.056
HDL-cholesterol (mmol/L), median (IQR)	1.14 (0.93, 1.36)	1.14 (0.93, 1.37)	0.911
LDL-cholesterol (mmol/L), median (IQR)	2.7 (2.05, 3.28)	2.67 (2.07, 3.15)	0.478
Fibrinogen (g/L), median (IQR)	3.0 (2.40, 3.51)	3.04 (2.47, 3.60)	0.55
D-dimer (mg/L), median (IQR)	0.42 (0.22, 1.0)	0.54 (0.28, 1.28)	0.05
Urine specific gravity, median (IQR)	1.02 (1.01, 1.02)	1.02 (1.02, 1.03)	0.308
NLR, median (IQR)	3.57 (2.17, 6.01)	3.12 (2.13, 4.99)	0.105
PLR, median (IQR)	149.68 (104.41, 204.94)	138.72 (107.45, 184.97)	0.398
LMR, median (IQR)	3.59 (2.45, 4.79)	3.04 (2.13, 4.14)	**0.004** ^∗^
MHR, median (IQR)	0.38 (0.26, 0.54)	0.43 (0.32, 0.63)	**0.003** ^∗^
Imaging date			
TOAST			0.664
LAA	76 (51.7%)	151 (45.8%)	
SAA	40 (27.2%)	112 (33.9%)	
CE	12 (8.2%)	29 (8.8%)	
UE	8 (5.4%)	16 (4.8%)	
OE	11 (7.5%)	22 (14.9%)	
Lesion location, *n*%			**0.039** ^∗^
Anterior circulation	113 (76.9%)	222 (67.3%)	
Posterior circulation	34 (23.1%)	108 (32.7%)	
Leukoaraiosis			0.595
0	22 (14.9%)	54 (16.4%)	
1	65 (44.2%)	150 (45.5%)	
2	35 (23.8%)	61 (18.5%)	
3	25 (17.0%)	64 (19.4%)	
Outcome			
Discharge NIHSS, median (IQR)	5.0 (2, 8)	1.0 (0, 3.25)	**<0.001** ^∗^
3 mo MRS			**<0.001** ^∗^
0	21 (14.3%)	152 (46.1%)	
1	31 (21.1%)	78 (23.6%)	
2	12 (8.2%)	33 (10.0%)	
3	25 (17.0%)	27 (8.2%)	
4	40 (27.2%)	35 (10.6%)	
5	12 (8.2%)	2 (0.6%)	
6	6 (4.1%)	3 (0.9%)	

PI: progressive infarction; NIHSS: National Institute of Health Stroke Scale; MRS: modified Rankin scale; 3 mo: 3 months; WBC: white blood cell; RBC: red blood cell; HGB: hemoglobin; PLT: blood platelet; PT: prothrombin time; BUN: blood urea nitrogen; Cr: creatinine; UA: uric acid; HDL: high-density lipoprotein; LDL: low-density lipoprotein; GSP: glycated serum protein; TOAST: Trial of Org 10172 In Acute Stroke Treatment; LAA: large artery atherosclerosis; SVO: small vessel occlusion; CE: cardioembolism; UE: undetermined etiology; OE: other etiology; IQR: interquartile range; SD: standard deviation. ^∗^*P* < 0.05.

**Table 2:** Evaluation of the effect of LMR on PI using multivariate logistic regression models.

	OR	95% CI	*P*
Adjusted model^1^			
LMR Q1 (reference)			
Q2	0.997	0.553-1.798	0.991
Q3	1.326	0.743-2.364	0.34
Q4	2.144	1.211-3.796	**0.009** ^∗^
Adjusted model^2^			
LMR Q1 (reference)			
Q2	1.059	0.579-1.939	0.852
Q3	1.436	0.794-2.597	0.231
Q4	2.362	1.326-4.208	**0.004** ^∗^
Adjusted model^3^			
LMR Q1 (reference)			
Q2	1.135	0.608-2.120	0.691
Q3	1.578	0.851-2.927	0.147
Q4	2.598	1.405-4.802	**0.002** ^∗^

^1^The adjusted model was controlled for age and male. ^2^The adjusted model was controlled for hypertension, initial NIHSS, Cr, fasting glucose, and lesion location. ^3^The adjusted model was controlled for hypertension, initial NIHSS, Cr, fasting glucose, lesion location, BUN, uric acid, TOAST, and leukoaraiosis. OR: odds ratio; CI: confidence interval; PI: progressive infarction; LMR: lymphocyte-to-monocyte ratio; NIHSS: National Institute of Health Stroke Scale; Cr: creatinine; BUN: blood urea nitrogen; TOAST: Trial of Org 10172 In Acute Stroke Treatment.

**Table 3:** Evaluation of the impact of LMR on PI in subgroups using multivariate logistic regression models.

	OR	95% CI	*P*
LAA (*n* = 227)			
Adjusted model^1^			
LMR Q1 (reference)			
Q2	1.007	0.440-2.305	0.987
Q3	1.280	0.571-2.872	0.549
Q4	2.335	1.051-5.191	**0.037** ^∗^
SVO (*n* = 152)			
Adjusted model^2^			
LMR Q1 (reference)			
Q2	1.195	0.330-4.324	0.786
Q3	1.067	0.314-3.618	0.918
Q4	1.911	0.585-6.250	0.284

^1^The adjusted model was controlled for fasting glucose, hypertension, initial NIHSS, and LDL-cholesterol. ^2^The adjusted model was controlled for uric acid, total cholesterol, triglyceride, LDL-cholesterol, D-dimer, and PT. OR: odds ratio; CI: confidence interval; LAA: large artery atherosclerosis; SVO: small vessel occlusion; LMR: lymphocyte-to-monocyte ratio; PT: prothrombin time.

**Table 4:** Evaluation of the association between LMR and PI based on the cutoff value.

	PI (%)	Non-PI (%)	*P*
All cases (*n* = 477)	147	330	**<0.001** ^∗^
LMR < 3.506 (*n* = 280)	69 (24.6%)	211 (75.4%)	
LMR ≥ 3.506 (*n* = 197)	78 (39.6%)	119 (60.4%)	
LAA (*n* = 227)	76	151	**0.001** ^∗^
LMR < 3.944 (*n* = 149)	39 (26.2%)	110 (73.8%)	
LMR ≥ 3.944 (*n* = 78)	37 (47.4%)	41 (52.6%)	

PI: progressive infarction; LMR: lymphocyte-to-monocyte ratio; LAA: large artery atherosclerosis.

## Data Availability

Data is available on request from the authors.
